# Protein Delivery into Plant Cells: Toward *In vivo* Structural Biology

**DOI:** 10.3389/fpls.2017.00519

**Published:** 2017-04-19

**Authors:** Cesyen Cedeño, Kris Pauwels, Peter Tompa

**Affiliations:** ^1^VIB Structural Biology Research Center, Vlaams Instituut voor BiotechnologieBrussels, Belgium; ^2^Structural Biology Brussels, Vrije Universiteit BrusselBrussels, Belgium; ^3^Institute of Enzymology, Research Centre for Natural Sciences of the Hungarian Academy of SciencesBudapest, Hungary

**Keywords:** *in-cell* NMR, fluorescence microscopy, electroporation, intrinsically disordered proteins, dehydrins, ERD14, ERD10

## Abstract

Understanding the biologically relevant structural and functional behavior of proteins inside living plant cells is only possible through the combination of structural biology and cell biology. The state-of-the-art structural biology techniques are typically applied to molecules that are isolated from their native context. Although most experimental conditions can be easily controlled while dealing with an isolated, purified protein, a serious shortcoming of such *in vitro* work is that we cannot mimic the extremely complex intracellular environment in which the protein exists and functions. Therefore, it is highly desirable to investigate proteins in their natural habitat, i.e., within live cells. This is the major ambition of *in-cell* NMR, which aims to approach structure-function relationship under true *in vivo* conditions following delivery of labeled proteins into cells under physiological conditions. With a multidisciplinary approach that includes recombinant protein production, confocal fluorescence microscopy, nuclear magnetic resonance (NMR) spectroscopy and different intracellular protein delivery strategies, we explore the possibility to develop *in-cell* NMR studies in living plant cells. While we provide a comprehensive framework to set-up *in-cell* NMR, we identified the efficient intracellular introduction of isotope-labeled proteins as the major bottleneck. Based on experiments with the paradigmatic intrinsically disordered proteins (IDPs) Early Response to Dehydration protein 10 and 14, we also established the subcellular localization of ERD14 under abiotic stress.

## Introduction

**Figure 1:**
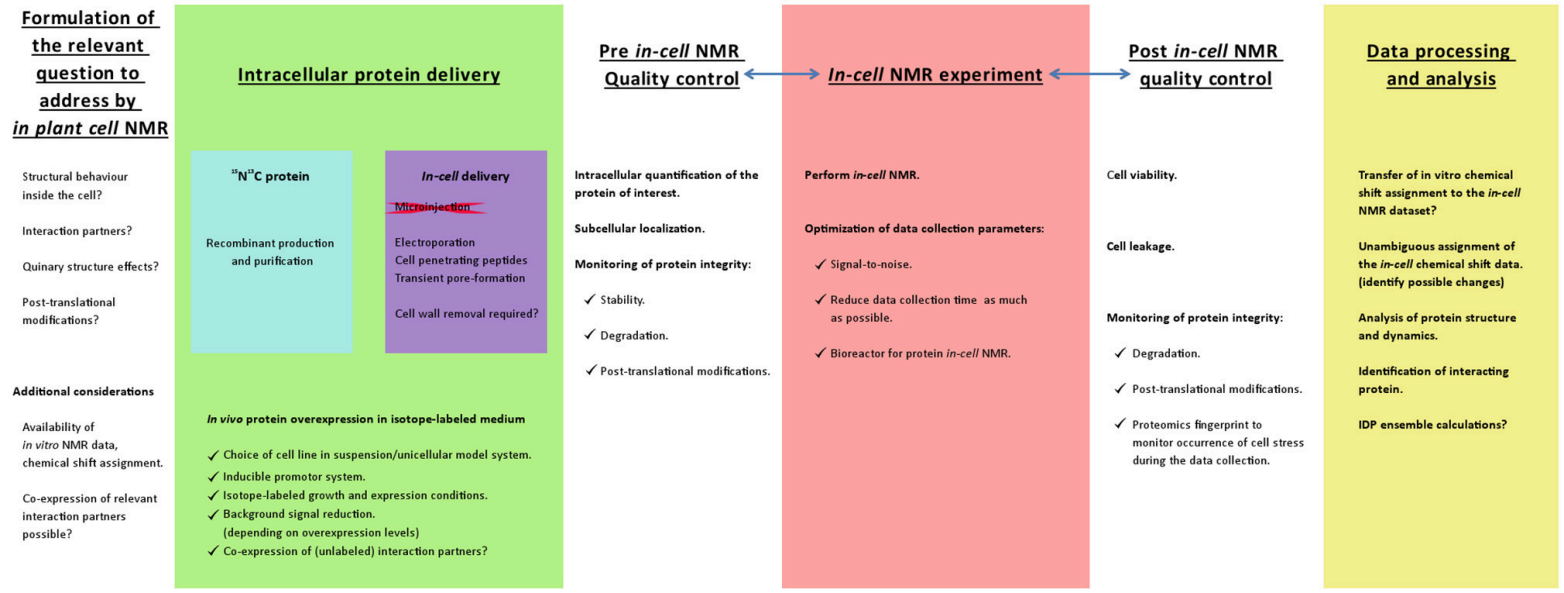
**Schematic work flow representation to develop an *in-cell* NMR strategy to study proteins within plant cells**. The first considerations and planning of the *in-cell* NMR experiments should be based on readily available NMR data. It is desirable that the protein of interest has been characterized *in vitro* by NMR and that a chemical shift assignment is available that ultimately can be used to analyze the *in-cell* NMR dataset. Since NMR is a high resolution technique, it can address specific questions with regard to structural and dynamical aspects at the residue-level of the protein intracellular behavior. The major experimental obstacle will be the intracellular delivery of isotope-labeled protein. The success of the delivery strategy should entice monitoring the subcellular localization, intracellular protein quantification and the protein integrity. These are also 3 important parameters to check upon completing the actual *in-cell* NMR data acquisition. Likewise, it is imperative to verify the cell viability and to demonstrate that the protein has not leaked into the extracellular medium so that the NMR signals truly originate from the intracellular protein (Waudby et al., 2012). Another aspect to consider is the occurrence of stress to the cells during the data acquisition, which might be unveiled by an in depth proteomics analysis. To maintain the plant cell suspension in a living state, the experiment can be performed in a bioreactor that was developed for *in-cell* protein NMR. The performance and capabilities of such a bioreactor has been demonstrated by the overexpression of the intrinsically disordered human protein α-synuclein in *Escherichia coli* (Sharaf et al., 2010). Ultimately the *in vitro* NMR assignment should be transferred to the *in-cell* NMR datasets and employed to answer the relevant questions.

When we want to study the conformations of plant proteins, their interactions and their functions in their native intracellular localization, we need to rely on a combination of molecular biophysics and cell biology. The conventional structural biology approaches that aim to elucidate the structure of proteins, such as X-ray crystallography and nuclear magnetic resonance (NMR), traditionally rely on samples of isolated, stable and folded proteins. These samples are the product of elaborate and sometimes tedious purification protocols. At the end, a homogeneous and highly concentrated protein sample usually yields a reliable and accurate description of its structural behavior. Solution-state biomolecular NMR offers an orthogonal approach to crystallographic methods, because the final experiment is not done in solid state, but with a protein that freely diffuses in an aqueous environment. Despite sample limitations in terms of the size, solubility and stability of the protein, NMR does not provide a single structural snapshot in the solid state, rather it provides comprehensive insight into the fully dynamic and flexible state of the protein that is much closer to its real functional existence (Dyson and Wright, 2004). A more realistic picture about life at the molecular level requires the observation of protein behavior as it happens in the cell. *In-cell* NMR is one of the techniques par excellence for this purpose. It often requires the intracellular delivery of isotopically labeled protein under conditions compatible with life, which can be accomplished with induced expression, microinjection or electroporation. There are well-documented protocols and insightful reports of proteins being studied inside mammalian cells, yeast and bacteria (Bekei et al., 2012a,b,c). To the best of our knowledge, there is no precedence of *in-cell* NMR experiments in plants. Samples for solution-state NMR (and thus also for *in-cell* NMR) should fit into a quite narrow tube, which is then placed inside a spectrometer were subtle magnetic field perturbations can be recorded. Among all types of plants and tissues with distinct cellular morphologies, only cells in suspension are suitable for scrutiny when studying proteins via *in-cell* NMR. This contrasts with the convenient and open framework that, for instance, microscopy can offer, yet the high resolution information obtained via *in-cell* NMR has a unique value. Since NMR spectroscopy is an inherently low-sensitivity technique, it requires a relatively high protein concentration (in the range of 10^−6^–10^−3^ M) for collecting reliable information. Such high concentrations for a given protein are not always incompatible with normal physiology. Therefore, only proteins that are abundant in cells are eligible for such *in-cell* NMR studies. In addition, only isotopically labeled proteins (^15^N,^13^C) are detected during the NMR experiment. Hence, the protein that will be studied inside cells should be labeled with these magnetically detectable isotopes. Several types of NMR experiments can then be carried out, for example, carbon detection (Hsu et al., 2009) provides an approach which is not sensitive to chemical exchange of protons in the amide groups, i.e., internal pH. This imposes a clear set of conditions: (a) the protein of interest must be obtained in a pure and isotopically labeled form and then introduced into host plant cells, or (b) the protein has to be over-expressed in plant cells under labeling conditions (in a growth medium containing isotopes), preferably under the control of a strong promoter (Figure 1). Either strategy has advantages and disadvantages. Yet, producing the protein exogenously (e.g., recombinant expression in *Escherichia coli* under isotope-labeling conditions followed by purification) in combination with a controlled delivery into plant cells, offers the most diverse palette of options and techniques for the purpose of *in-cell* NMR. Recombinant production in *E. coli* often constitutes simple and robust protocols that lead to a sample that is devoid of post-translational modifications. This can be a drawback when the post-translational modification is essential for the protein functionality, but also offers the opportunity to study the post-translational modification inside the plant cell.

Historically, different techniques have been used to deliver macromolecules (DNA and polypeptides) across the membranous boundaries of cells. The cell membrane can be transiently disrupted by either chemical or physical treatment, causing pore formation that allows the diffusion of material from outside the cell to its interior. Chemical manipulation of cells yielding permeabilization and delivery of materials involves the use of Ca^2+^/Mg^2+^, Ca^2+^/PEG, PEG/DMSO, detergents, toxins (e.g., streptolysin) or cell-penetrating peptides (Hanahan, 1983). This has proven to be a convenient way for transforming *E. coli* cells with foreign plasmid DNA. In this regard, plant cells pose a special challenge due to the presence of a glycan-rich cell wall. This can be overcome by either adjusting experimental conditions or removing the cell wall (creating protoplasts) by enzymatic degradation (Vanden Bossche et al., 2013). Physical manipulation of cells can rely on the use of strong mechanical forces, which is the case of microinjection or cell bombardment, or via the use of electric field pulses in the case of electroporation (Jaffe and Terasaki, 2004; Kikkert et al., 2005; Chen et al., 2006). Micromanipulators and micro-injecting systems, which usually require a dedicated microscope and a cell sorter, have been used with success to deliver and study proteins inside cells (König et al., 2015). Cell size plays an important role here, as in most cases micro-injection has succeeded with very large cells, such as *Xenopus laevis* oocytes (Thongwichian and Selenko, 2012). Cell bombardment has also been used successfully for DNA delivery into plant cells and tissues. However, success with this technique is limited by the amount of material that can be loaded on a particle and the ratio of surviving cells. For our study, we have opted for electroporation to deliver the protein of interest into plant cells. To develop our methodology (Figure 1), we have selected tobacco BY-2 cells (a plant cell line derived of *Nicotiana tabacum* cv Bright Yellow 2) as model system (Nagata et al., 1992, 2004). This frequently used plant cell model grows in suspension, it is easy to maintain and these cells are amenable for *in vivo* imaging by wide-field and confocal laser microscopy. BY-2 cells can also be manipulated to test different protocols for protein delivery among the repertoire described above (Nagata et al., 2004). Finally, we used intrinsically disordered proteins (IDPs) that are generally good candidates for *in-cell* NMR studies due to the high peak intensity and good signal dispersion they exhibit at low concentration. Despite the small ranges of chemical shifts attained in spectra of IDPs, the dispersion of the NMR signals in IDPs is favored by their extremely long relaxation times leading to very narrow resonances, which makes the resolution of their peaks highly likely. ERD14 and ERD10 are such IDPs that belong to the class of dehydrins, a subgroup of Late Embryogenesis Abundant (LEA) proteins (Hundertmark and Hincha, 2008). LEA proteins are commonly found in higher plants (Battaglia et al., 2008), but they also occur in small rotifers (Chakrabortee et al., 2012). Originally discovered as cotton-seed abundant proteins, there is increasing evidence that they occur in different plant tissues and developmental stages (Candat et al., 2014). Dehydrins are of particular interest as they exhibited chaperone-like behavior while being intrinsically disordered (Kovacs et al., 2008a). Experimental data about the actual mechanism of their action *in vivo* is missing. In this contribution we explore the suitability of these two proteins as molecular models for *in-cell* NMR in plant cells.

## Materials and methods

### Cloning of LEA proteins ERD14 and ERD10

#### Cloning of erd14 and erd10 into gateway™ vectors

For Red Fluorescence Protein (RFP) fusions cloning was done according to standard procedures with existing *erd14* and *erd10* fusion constructs (Kovacs et al., 2008b) which were subsequently amplified and recombined with a pDONR221 vector (Invitrogen GatewayTM cloning kit, Cat. No. 12535 029) according to the manufacturer's instructions yielding both *erd14* and *erd10* pENTR vectors without stop codon suitable for recombination with pK7WGR2.0 (an in-frame C-terminal RFP GatewayTM vector (Karimi et al., 2007). Reaction between attL1/L2 sites on the pENTR*erd14* (or *erd10*) and attR1/R2 sites on pK7WGR2.0 was carried out using LR clonase at 25°C during 1 h in 10 mM Tris.HCl, 1 mM EDTA, pH = 8.0 (TE buffer). Then proteinase K was added and incubated for 10 min at 37°C. Material was transformed into *E. coli* TOP10 chemically competent cells in order to replicate plasmid DNA that was purified for subsequent work using a Wizard Wizard R Plus SV Minipreps DNA Purification System. (Promega Corporation, Cat. No. A7510). Finally, both *erd14-rfp* and *erd10-rfp* fusion constructs were confirmed correctly in frame within pK7WGR2.0 by DNA sequencing.

#### Codon insertion for erd14 for fluorescent labeling via a cysteine residue

Restriction-free cloning allowed the introduction of cysteine-186 at the end of ERD14 coding region. This mutant was designed using a pET22b-ERD14 template vector employed to over-express and purify ERD14 from *E. coli*. The primers introducing a C186 mutation into ERD14 (ERD14-C186) are: ERD14-C186fw 5′-GAGGAGAAGAAAGATAAAGAATGTTAAGCGGCCGCACTCGCGCACCAC-3′ ERD14-C186rv 3′-CCTCCTCTTCTTTCTATTTCTTACAATTCGCCGGCGTGAGCGCGT-5′.

The PCR reaction for this cloning resembles a site-directed mutagenesis reaction but in this case a new codon is inserted at position 186. The PCR reaction mixture comprised 125 ηg of ERD14-C186_fw_ primer, 125 ng of ERD14-C186_rv_ primer and 50 ng of pET22b-ERD14wt template DNA, mixed with 12.5 μL of HiFi KAPA Hot Start ReadyMix (KAPA Biosystems) in a final volume of 25 μL. The PCR programme included 3 min of denaturation at 95°C followed by 25 cycles of 20-s denaturation at 98°C, 15-s annealing at 65°C and 80-s extension at 72°C. Finally, another extension of 6 min at 72°C was included. The reaction mixture was cooled down and 1 uL of DpnI was added in order to digest methylated DNA for 90 min at 37°C. The obtained material was transformed into *E. coli* DH5α Ca^2+^-competent cells and selected on LB-agar plates supplemented with carbenicillin. Finally, successful mutation was confirmed by DNA sequencing.

### Generation of a stable BY-2 cell line expressing ERD14/10-RFP

Generation of these cell lines involved several steps as described below.

#### Transformation of agrobacterium tumefaciens with the appropriate DNA constructs of ERD14/10

An aliquot of electrocompetent *A. tumefaciens* LBA4404 cells (50 μL) was transferred into a pre-cooled electroporation cuvette (Bio-Rad Laboratories Inc., Cat No. 165-2086) and mixed with 2 μL of DNA (pK7WGR2.0-ERD14-RFP or pK7WGR2.0-ERD10-RFP) ensuring that no air bubbles were present. Cells were shocked on a Gene Pulser XcellTM electroporation apparatus (Bio-Rad Laboratories Inc.) using the following settings: 2.5 kV, 400 MΩ, 25 F. Next, 300 μL of Super Optimal broth Catabolite repression medium (SOC; 20.0 g/L tryptone, 5.0 g/L yeast extract, 0.5 g/L NaCl) was added to the cuvette and the total volume was transferred to a 12 mL culture tube containing additional 1 mL of SOC medium5. This culture was incubated for 2 h at 28°C, while shaking at 180 rpm. Next, cells were spread onto Yeast Extract Broth (YEB) plates containing gentamycin (20 μg/mL), rifampicin (25 μg/mL) and bacterial selection antibiotic for the gene of interest (spectinomycin for K7WGR2.0-ERD14-RFP and K7WGR2.0-ERD10-RFP fusions). Colonies were detected after 48 h at 28°C and finally transferred into YEB liquid medium (5.0 g/L beef extract, 1.0 g/L yeast extract, 5.0 g/L peptone, 5.0 g/L sucrose, 0.5 g/L MgCl2) supplemented with appropriate antibiotics and grown for 48 h at 28°C for further use or storage as glycerol stocks.

#### BY-2 infection with positive clones of *A. tumefaciens*

A fresh BY-2 culture was prepared after diluting 1 mL of a 1 week old culture into 39 mL of fresh BY-2 medium (4.302 g/L Murashige-Skoog Salts; Murashige and Skoog, 1962) (Cat. No. M0301-0050, DUCHEFA Biochemie B.V.), 0.2 g/L KH2PO4, 30.0 g/L sucrose, 0.08 μg/L 2,4-dichlorophenoxyacetic acid (auxin synthetic analog), 1 μg/L Thiamine and 0.1 mg/L myo-inositol, equilibrated until pH = 5.8 using KOH). Upon incubation for 72 h, 3.7 mL of the fresh dilution was transferred into a Petri dish without antibiotics and mixed with 300 μL of transformed *A. tumefaciens*. The mix was placed for incubation for 48 h at 25°C, without shaking. The content of each transfection dish was spread onto BY-2 solid-medium plates supplemented with vancomycin, carbenicillin and the plant selection antibiotic (kanamycin in the case of pK7RWG2.0-ERD14 or pK7RWG2.0-ERD10). Positive calli were selected on these plates after 4 weeks at 25°C by confirming the expression of RFP using fluorescence microscopy. Positive calli were transferred to fresh plates every 3–4 weeks or to liquid BY-2 medium for imaging purposes.

### Generation of a stable cell line for overexpression of ERD14 and ERD10 in BY-2 and *A. thaliana*

Cloning of *erd14/10* with an inducible promoter region (regulated by estrogen) was performed using the same pENTR constructs obtained above. The destination vector pDEST contains a region for promoter binding upon induction and together with the gene of interest it was integrated into BY-2 facilitated by *A. tumefaciens* infection as described above. The expression profile for either ERD14 or ERD10 in BY-2 and also in *Arabidopsis* cells growing in suspension was monitored for a period of 96 h and detected by Western Blotting. The labeling medium for incorporating 15N isotopes into BY-2 was prepared by replacing the corresponding inorganic salts (ammonium sulfate and ammonium nitrate) in normal Murashige-Skoog media (Murashige and Skoog, 1962) with the salts incorporating the ^15^N isotope.

### Transient transformation of ERD10/14 into *Nicotiana benthamiana* leaves

For localization *in planta*, ERD14/10 was transiently transformed on *Nicotiana benthamiana* leaves. *A. tumefaciens* LBA4404 transformed with the *erd14-rfp* or *erd10-rfp* fusion gene was grown in 5 ml YEB medium, supplemented with the corresponding antibiotics at 28°C for 48 h shaking at 180 rpm. OD_600_ was measured and cultures were diluted to a final volume of 4 mL to an OD_600_ = 1.5 and spun down for 5 min at 4,000 g. The pellet was resuspended in 4 mL of infiltration buffer using conical-bottom 15 mL tubes (10 mM MgCl_2_, 10 mM MES buffer pH = 5.6 and 0.1 mM acetosyringone). Each tube was incubated for 2 h at room temperature in a tube rotator. Infection was performed by injecting 0.5 mL of the previously described mix on the underside of several *N. benthamiana* leaves using a syringe without needle (Li, 2011). Upon infection and incubation for 72 h, plants were investigated by confocal microscopy to verify RFP-fused protein production by cutting a section of a leave and placing it directly on a cover slip (section of 1 cm^2^ approx.).

### Production and purification of ERD14

*E. coli* BL21(DE3) cells were transformed with ERD14-C186 pDNA according to standard procedures and a positive colony was selected from an LB-agar plate supplemented with carbenicillin to prepare a pre-culture of 50 mL liquid NZYM medium (10.0 g/L NZ amine, 5.0 g/L NaCl, 5.0 g/L Bacto-yeast extract, 2.0 g/L of MgSO4.7 H2O) also supplemented with antibiotics that was grown overnight at 37°C while shaking at 180 rpm. The pre-culture was used to inoculate 2 L of NZYM contained in 2 independent baffled flasks each supplemented with 50 mg/mL carbenicillin, these culture flasks were incubated in an orbital shaker at 180 rpm and 37°C. After 2 h, the culture reached OD600 = 0.6; at this stage 500 μL of 1 mM isopropyl-β-D-1-thiogalactopyranoside (IPTG) was added and the cells continued shaking at the same speed and temperature for 4 h. The bacterial pellet was collected by centrifugation at 4,000 g and 4°C for 30 min and resuspended in 50 mL ice-cold lysis buffer (50 mM CAPS, 5 mM EDTA, 5 mM tris(2-carboxyethyl)phosphine (TCEP), 1 Roche complete inhibitor cocktail tablet at pH = 10). Cell lysis was performed on an ice-cold bath by sonication using a Sonics Vibra-Cell™ CV18 model ultrasonic processor (applying 60 power of the probe in 6 cycles of pulses, 15 s ON and 30 s OFF). The suspension was then cleared by centrifugation at 20,000 g for 45 min, and the supernatant was placed into a water bath at 100°C for 5 min. After boiling, the solution was cleared by centrifugation at 20,000 g for 45 min and the supernatant was filtered through a 0.5 μm filter before injection on a QFF HiTrap 5 mL column (GE Healthcare) for anionic exchange. Binding buffer (50 mM CAPS, 5 mM TCEP, pH = 10) was used to equilibrate the column and the supernatant was passed through the column at 2 mL/min flow using an AKTA Pure system. While the ultraviolet (UV) absorbance at 215 nm was used to monitor protein elution. Weakly bound proteins were washed out with 5 column volumes (CV) of 2% elution buffer (50 mM CAPS, 250 mM NaCl, 5 mM TCEP, pH = 10) before starting a linear gradient between 2 and 50% (using same elution buffer) without changing the flow. Fractions containing the protein of interest were identified by SDS-PAGE (detection with PageBlueTM protein staining solution, ThermoScientificTM, Cat. No. 24620) and concentrated using 20 mL spinning filters with a 3 kDa cut-off (Vivaspin 20, Sartorius AG). The concentrated sample containing ERD14-C186 and some minor contaminants was injected onto a Superdex75 16/100 HiLoad column (GE Healthcare) and the gel filtration buffer consisted of phosphate buffered saline (PBS) pH = 7.5 supplemented with 5 mM TCEP). Fractions were collected automatically based on the UV absorbance profile and SDS-PAGE allowed the identification of the protein of interest at the corresponding retention time. Selected fractions were concentrated above 150 μM for use in labeling reactions.

### Fluorescent labeling of ERD14 by Alexa Fluor 488 and Alexa Fluor 647

Alexa Fluor® 488 C5 Maleimide (green) and Alexa Fluor® 647 C2 Maleimide Alexa Fluor 647 (red) were purchased as lyophilized salts from ThermoScientificTM, Cat.No.A10254 and Cat.No.A20347, respectively. A HiTrap Desalting 5 mL column (GE Healthcare) was used to remove TCEP from the protein sample, to which either Alexa488 maleimide or Alexa Fluor 647 maleimide reagents were immediately added. The reaction mixtures (5-fold excess of fluorescent dye compared to protein concentration) were placed on a thermal bath at 60°C for 1 h. Labeling of ERD14-C186 was confirmed by SDS-PAGE through the presence of a fluorescent band with the molecular size corresponding to an ERD14 monomer. An additional gel filtration step was performed to separate the labeled protein from disulfide-linked dimers and unreacted fluorophores.

### Electroporation experiments for protein delivery

Electroporation experiments were performed on a Gene Pulser MXCellTM Electroporation System using 2 mm cuvettes (Bio-Rad Laboratories Inc., Cat No. 165-2086). A volume of 40 μL of non-modified BY-2 cells was placed inside the cuvette together with 10 μL of 250 μM ERD14-C186-Alexa488 in PBS. A broad range of conditions were tested in order to assess protein uptake and cell survivability. These conditions are summarized in Table 1 in the Results section. In every case, cells were electroporated in BY-2 medium and cargo protein ERD14-C186-Alexa488 or ERD14-C186-Alexa Fluor 647 was added in PBS. Cells were collected from the cuvette and resuspended in 100 μL of BY-2 medium. At this stage, cells were allowed to recover for different periods of time before imaging with either confocal or epifluorescence microscopy.

**Table 1:** **Scouting of various electroporation conditions**.

	**Experimental condition**											
**Waveform**	**1**	**2**	**3**	**4**	**5**	**6**	**7**	**8**	**9**	**10**	**11**	**12**
Exponential	150 V	200 V	250 V	300 V	350 V	450 V	250 V	250 V	250 V	250 V	250 V	250 V
decay	350 μF	350 μF	350 μF	350 μF	350 μF	350 μF	200 μF	250 μF	350 μF	500 μF	750 μF	1,000 μF
	400 V	250 V	150 V	100 V	50 V	50 V	150 V	150 V	150 V	150 V	50 V	50 V
	200 μF	200 μF	200 μF	200 μF	200 μF	100 μF	250 μF	500 μF	750 μF	1,000 μF	500 μF	750 μF
Square	150 V	200 V	250 V	300 V	350 V	450 V	250 V	250 V	250 V	250 V	250 V	250 V
wave	20 ms	20 ms	20 ms	20 ms	20 ms	20 ms	5 ms	10 ms	15 ms	20 ms	25 ms	30 ms

*The electric pulse is a wave with an associated voltage, capacitance and time. There are two different waveforms, namely square wave or exponential decay. These experimental conditions were tested to load BY-2 cells with externally supplied ERD14-C186-Alexa Fluor 647 as reporter protein. We monitored the cell integrity and cell viability as a biological read-out through microscopic observations*.

### Confocal and epifluorescence microscopy

Cell viability was inspected by mixing electroporated cells with either lipophilic styryl dye (FMTM Thermo Fisher Scientific Inc., Cat. No. F35355) or propidium iodide (PI, Thermo Fisher Scientific Inc., Cat.No.P3566) directly on the cover slide. For cell culture monitoring and calli selection, an inverted epifluorescence microscope (ZEISS Axiovert 135M) was used. Excitation is done with an HBO 100 Hg lamp. Images were captured with a ZEISS Axiocam using the software package axiovision 4.8.2 SP2 (06-2012). Using single GFP filter and RsGFP ex 465-490 nm; em 500–520 nm. Lenses used are either plan-apochromat 20x dry na = 0.75 or plan apochromat 40x dry na = 0.95. In confocal imaging a LSM5 Exciter upright microscope using an AxioImager Z1 microscopy stand was used. Different lenses were used and are described as follows: plan-apochromat 20x dry na = 0.8, C-apochromat 63x water corrected na = 1.2, C-apochromat 40x water corrected na = 1.2. In every case imaging using dual GFP/RFP scanning was performed using software package ZEN2009.

### Solution state NMR experiments

NMR experiments (SOFAST HN-HSQC; heteronuclear single quantum coherence) (Schanda et al., 2005) were performed on a 750 MHz Bruker250 Avance spectrometer equipped with a cryogenically cooled triple resonance 1H{13C/15N} TCI probe. Samples were loaded into 3 mm Shigemi tubes and supplemented with 10% D2O. Spectra of samples in buffer were collected using 128 scans and 1,024 increments while samples in extract were collected using 256 scans and 2,048 increments. Samples recorded either for reference (in buffer) or in cell lysate were 25 μM ERD14 or ERD10 in 50 mM MES buffer; pH = 6.5. Crude Extracts (cell lysates) were obtained by sonicating 4 days old BY-2 cells on an ice-cold bath using a Sonics Vibra-CellTM CV18 model ultrasonic processor (applying 60 power of the probe in 2 cycles of pulses, 10 s ON and 20 s OFF). Cells were suspended in 50 mM MES buffer in presence of 10 mM DTT, supplemented by cOmplete protease inhibitor and PhosSTOP phosphatase inhibitor. Extracts were not centrifuged or cleared of debris. The total protein concentration was determined by the adjusted Bradford assay for NanoDropTM 2000c according to the technical note from the manufacturer.

### Western blotting

Western blotting was performed using secondary antibodies at two different wavelengths, namely IRDye 680LT Goat anti-Mouse IgG for 680 nm and IRDye 800CW Goat Anti-Rabbit IgG for 800 nm. Imaging of developed membranes was done using dual-wavelength scanning using a LiCor Odessey® CLx. The primary antibody against ERD10, affinity purified anti-K segment antibody (Agrisera, Cat. No. AS07-206A).

## Results

### Scientific questions to be addressed by *in-cell* NMR

It is reported that both endogenous ERD14 and ERD10 are over-expressed *in planta* under abiotic stress, like low temperature and drought (Hundertmark and Hincha, 2008). The quest for the structural and functional characterization of ERD14 relied on *in vitro* techniques, including NMR, and revealed that the protein is intrinsically disordered (Kovacs et al., 2008b; Szalainé Ágoston et al., 2011). The NMR chemical shift assignment for ERD14 is already reported (BMRB entry 26636) and further calculations supported the idea that stable secondary-structure elements are not present (Close, 1996; Szalainé Ágoston et al., 2011). The NMR assignment of ERD10 was recently made publicly available by our group (Cedeño et al., 2017; BMRB entry 26949). The availability of the chemical shift assignment for both proteins is a prerequisite when evaluating the feasibility to conduct *in-cell* NMR experiments (Figure 1). With such an *in-cell* NMR approach, it becomes possible to probe if the *in vitro* determined structural and dynamical properties actually reflect the cellular *in vivo* conformation (Selenko and Wagner, 2007). Other interesting questions relate to the identity and structural influence of binding partners, the effect of post-translational modifications on protein conformation and the ways the proteins respond structurally to cellular processes like cell differentiation. To address the high resolution conformational behavior of the ERD proteins in response to external environmental changes (e.g., abiotic stress), the development of an *in-cell* NMR methodology is indispensable.

### *In vitro* NMR under molecular crowding conditions

**Figure 2:**
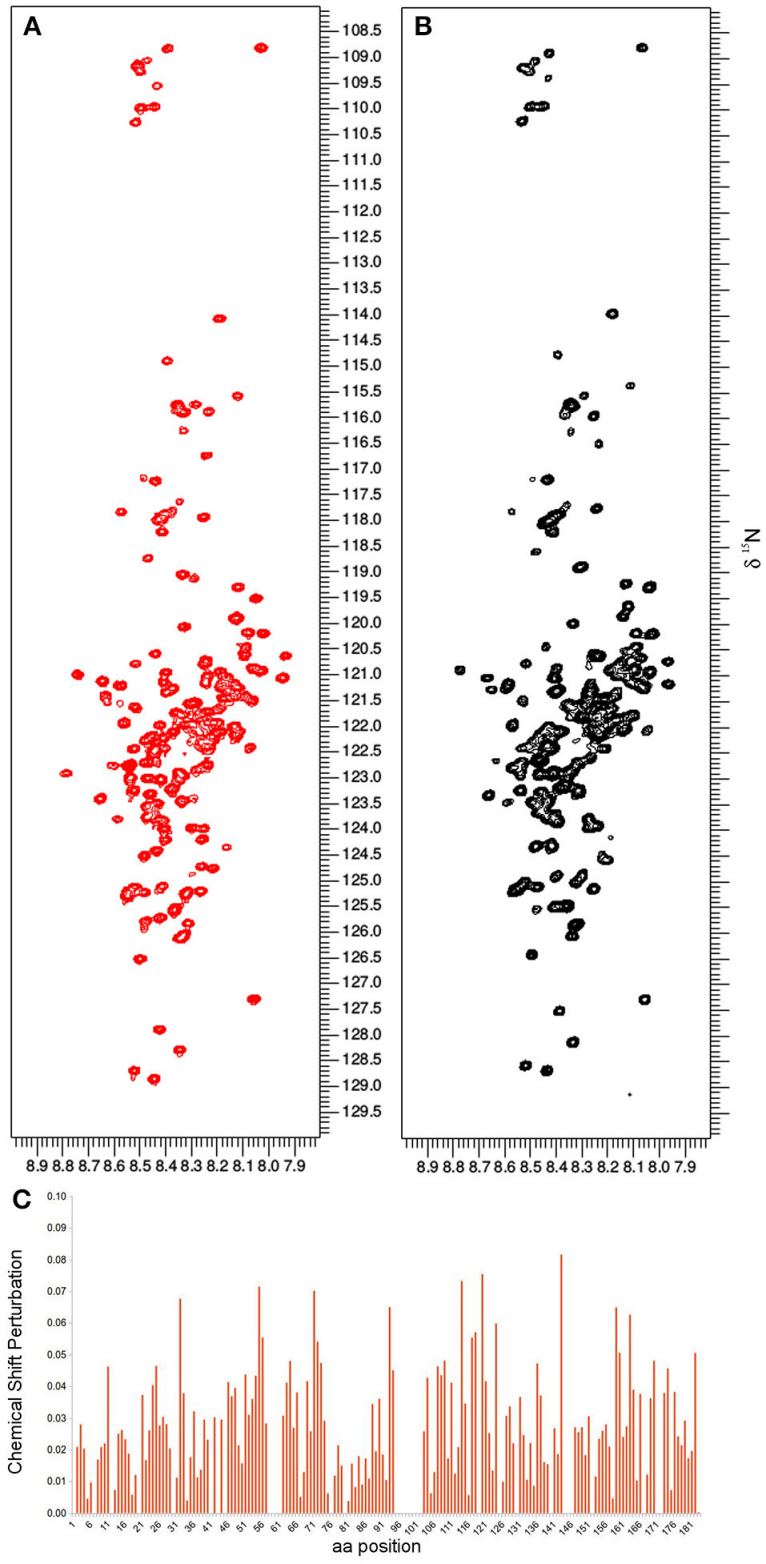
**The *in vitro* NMR analysis for ERD14 comprised the collection of ^1^H-^15^N-HSQC spectra and monitoring the chemical shift perturbation (CSP)**. ^1^H-^15^N-HSQC spectra were obtained with 25 μM of ^15^N-labeled ERD14 in 50mM MES buffer at pH = 6.5 **(A)** and under molecular crowding conditions that were generated with a cell lysate of BY-2 cells **(B)**. The CSP for ERD14 is represented in **C** and reveals changes in the chemical shifts of the protein due the modification of its environment. These CSP data can typically be used to determine the location of binding sites, the affinity of interactions or to monitor conformational changes of the complex.

**Figure 3:**
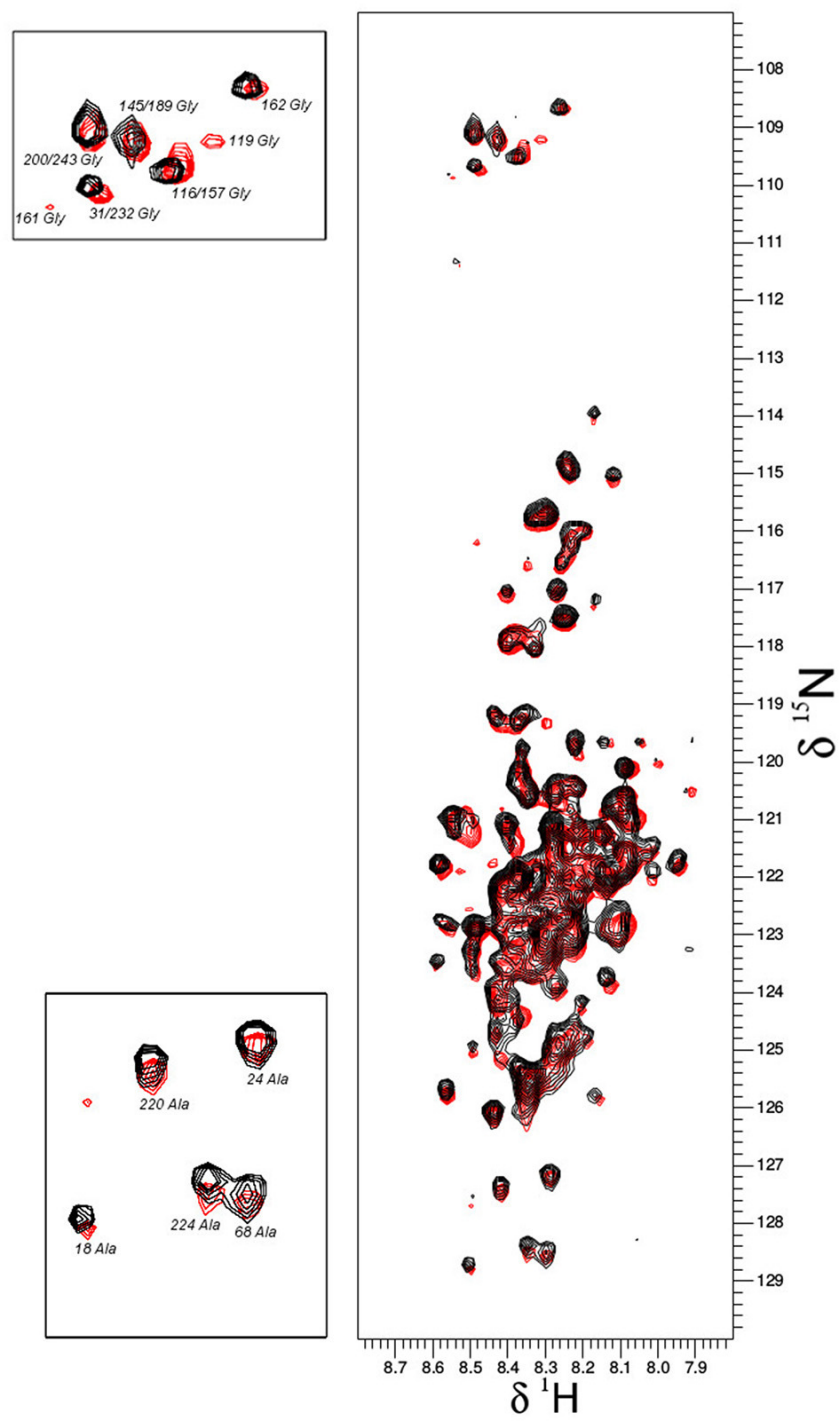
**Overlay plot of the NMR ^1^H-^15^N-HSQC spectra of ^15^N-labeled ERD10 that were collected under two different *in vitro* conditions**. The ^1^H-^15^N-HSQC spectrum of 25 μM ERD10 in 50 mM MES buffer pH = 7.3 (red) is superimposed with the ^1^H-^15^N-HSQC spectrum of 25 μM ERD10 that was measured in a BY-2 cell extract (black). The upper-left panel shows the area in the HSQC spectrum were most glycines are overlapped, while the lower-left panel shows well separated alanines in greater detail. No significant chemical shift perturbation is observed when comparing the two datasets.

Based on the already available NMR data, we opted to use the same experimental set-up, including the slightly acidic pH of 6.5 that limits the amide-proton exchange (Szalainé Ágoston et al., 2011). When we studied both the purified ERD14 and ERD10 by *in vitro* NMR, regardless of their size, we observed a relatively low overlap of the cross-peaks in their ^15^N-^1^H-HSQC spectra (see Figures 2A, 3). Besides these NMR experiments in a defined buffer, we also collected^15^N-^1^H-HSQC spectra of purified recombinant ^15^N-labeled ERD14 and ERD10 that were added to a cell extract of BY-2 to mimic the intracellular environment (Figures 2B, 3). Although the total protein concentration in cell extracts is far from that existing in the cytoplasm, this approach provides a reasonable and often applied approximation of cellular conditions, interaction partners and metabolites, interfering with the protein. Macromolecular concentrations closer to those prevailing in the cell can be achieved by the application of polymers, such as polethylene-glycol or dextran, which does not capture specific interactions with the protein, though. Overall, we could visualize some effects of crowding, as evidenced by line broadening for both ERD protein samples. These spectra showed little changes due to local changes of pH along the sequence as can be evidenced by the chemical shift changes experienced by histidines, which are particularly sensitive to this. It is conceivable that ERD14 and ERD10 directly interact with other cellular proteins and/or components in the crude lysates, but the data shown in this contribution do not allow us to detect such interactions (Cedeño et al., 2017). Additionally we did not observe signs in the HSQC spectra that would indicate degradation of the ^15^N-labeled proteins during the sample preparation and NMR data acquisition. From these experiments we concluded that the NMR data of the ERD proteins under molecular crowding conditions that resemble the plant cell interior contain sufficient information to initiate further *in-cell* NMR studies.

### Intracellular localization of dehydrins ERD14 and ERD10

**Figure 4:**
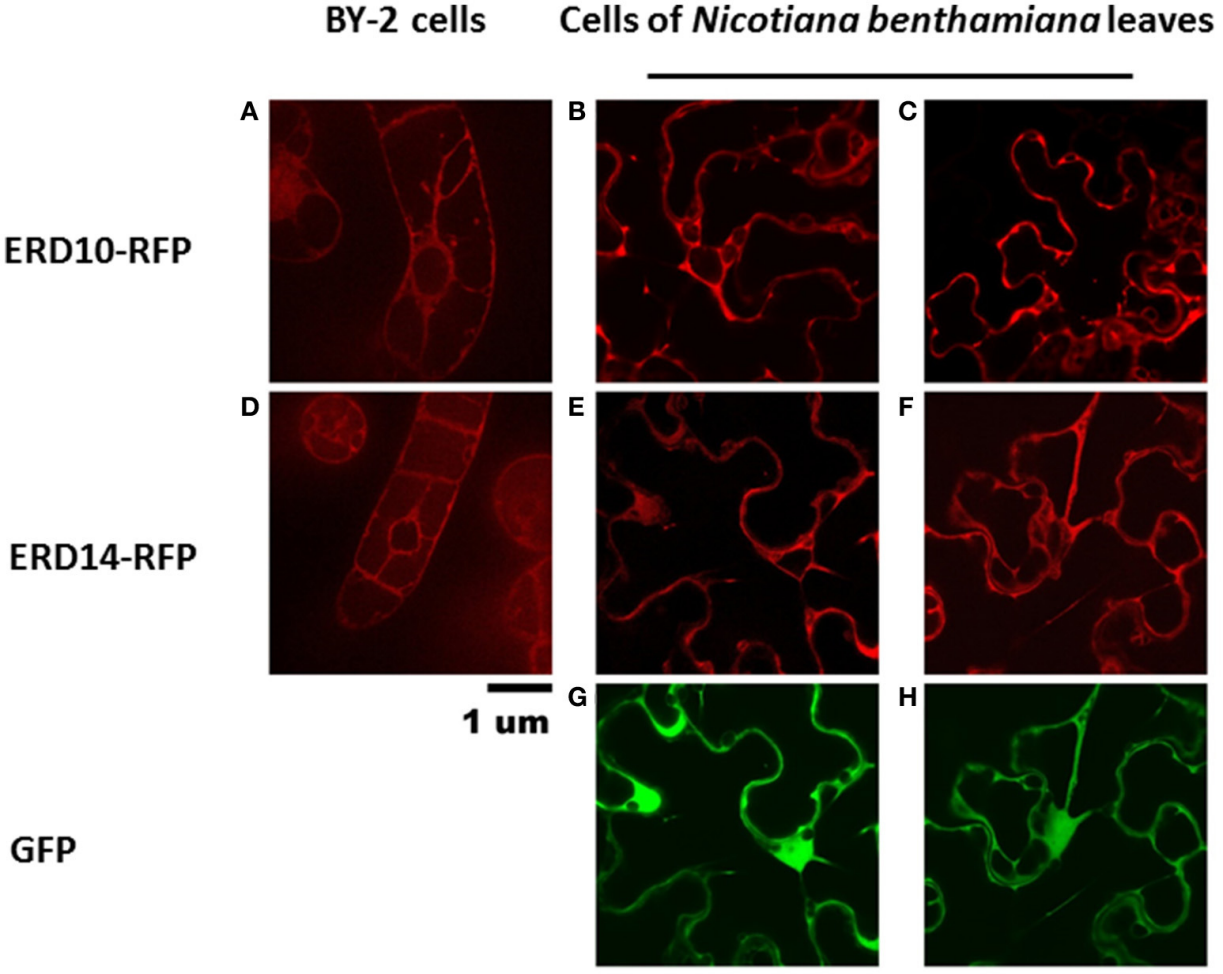
**Localization of ERD10-RFP, ERD14-RFP and green fluorescent protein (GFP) in BY-2 cells and in *N. benthamiana* leaves**. The ERD10-RFP fusion proteins are detected by fluorescence microscopy in the cytosolic strands in BY-2 cells **(A)** and in cells of *N. benthamiana* leaves **(B,C)**. ERD14-RFP fusion proteins are also observed in the cytosolic strands of BY-2 cells **(D)** as well as in cells of *N. benthamiana* leaves **(E,F)**. Control experiments were done using GFP **(G,H)** whereby dual channel images **(E,G,F,H)** are shown for the co-transfection of ERD14-RFP and GFP **(G,H)**.

**Figure 5:**
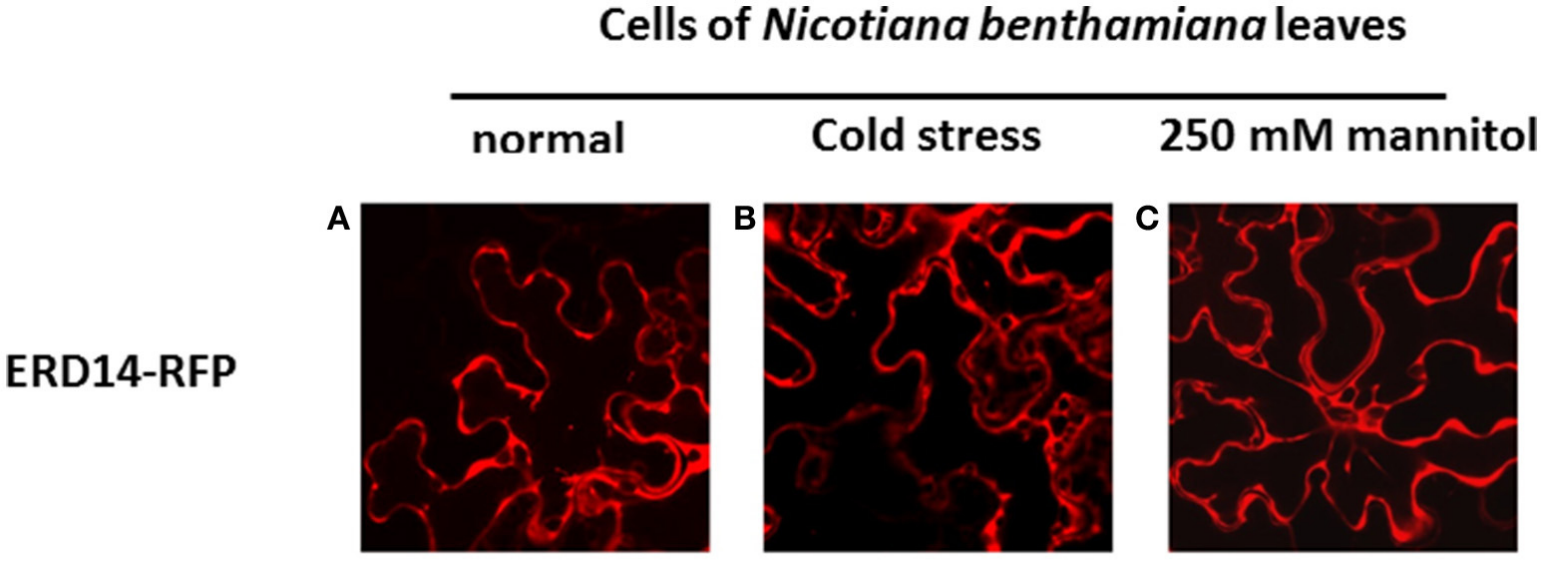
**Influence of abiotic stress on the subcellular localization of ERD14-RFP in *N*. *benthamiana* leaves based on fluorescence microscopy**. ERD14-RFP is located in the cytosolic strands in *N. benthamiana* leaves under normal conditions **(A)**. Under simulated stress conditions like cold **(B)** and hyperosmotic shock using 250 mM mannitol **(C)** the intracellular localization of ERD14-RFP remains unaltered.

To set the stage for monitoring intracellular delivery of ERD proteins, we set-out to explore the localization of ERD proteins in plant cells. Since the exact localization of ERD14 and ERD10 inside the plant cell remains elusive and debated (Close, 1996; Puhakainen et al., 2004), we created *erd10* and *erd14* gene-fusions with the Red Fluorescent Protein (RFP) that were introduced into BY-2 cells using *A. tumefaciens* transformants. Calli that exhibited the red fluorescence of ERD14-RFP or ERD10-RFP were selected under the epifluorescent microscope and then transferred to BY-2 liquid media in order to generate stable cell lines for each protein. As clearly seen in Figures 4A,D, ERD proteins exhibit a cytosolic localization under our experimental conditions. Although our observation contrasts with previous reports (on nuclear or chloroplast association), it is in agreement with a recently published independent study (Candat et al., 2014). The same protein fusionconstructs were used for transient expression on *N. benthamiana* leaves together with GFP as control. As can be seen in Figures 4B,E, the fusionproteins remain in the cytosolic strands, while co-expressed GFP migrates into the nucleus (Figure 4G). ERD10 was also transiently expressed on *N. benthamiana* leaves and showed similar localization (Figures 4C,F). GFP co-expression controls for ERD10 are shown in Figure 4H. We found that ERD14 does not change its localization upon induced cold stress and osmotic stress (that mimics dehydration). This suggests that the protective effect of this protein (Battaglia et al., 2008; Hundertmark and Hincha, 2008) is compatible with its cytosolic localization, as simulated on leaves of *N. benthamiana* (Figure 5, freezing is shown in Figure 5B, osmotic stress in Figure 5C). Altogether, these results suggest that ERD14 is a good candidate for *in-cell* NMR and that the protein is located in the cytoplasm.

### Electroporation for intracellular delivery of dehydrins

Because we could localize intracellular ERD14-RFP upon induced overexpression with success, and because of the quality of the NMR data of ERD14 under molecular crowding conditions, we decided to introduce purified and recombinantly produced 15N-labeled ERD14 inside plant cells using electroporation for *in-cell* NMR studies. We opted specifically for electroporation because DNA and small amounts of proteins can be delivered into plant cells by electroporation, it is possible to prepare samples containing 10^6^ cells in a matter of minutes/hours, and commercially available electroporators offer flexibility in varying the voltage and current as well as pulse timing and recovery rates (Yamano et al., 2013). These latter physical parameters determine efficient delivery of the material as well as survivability of the cells after the electric shock. Yet, our approach essentially differs from a transformation with DNA by electroporation strategy in some fundamental aspects: for transformation the main goal is to deliver a limited amount of DNA into a number of cells that must survive and get selected prior to further propagation. In contrast, for *in-cell* NMR, most of the cells should survive the procedure and maintain their (sub) cellular integrity, while most of the cells have to take-up a sufficiently large quantity of isotope-labeled protein material.

**Figure 6:**
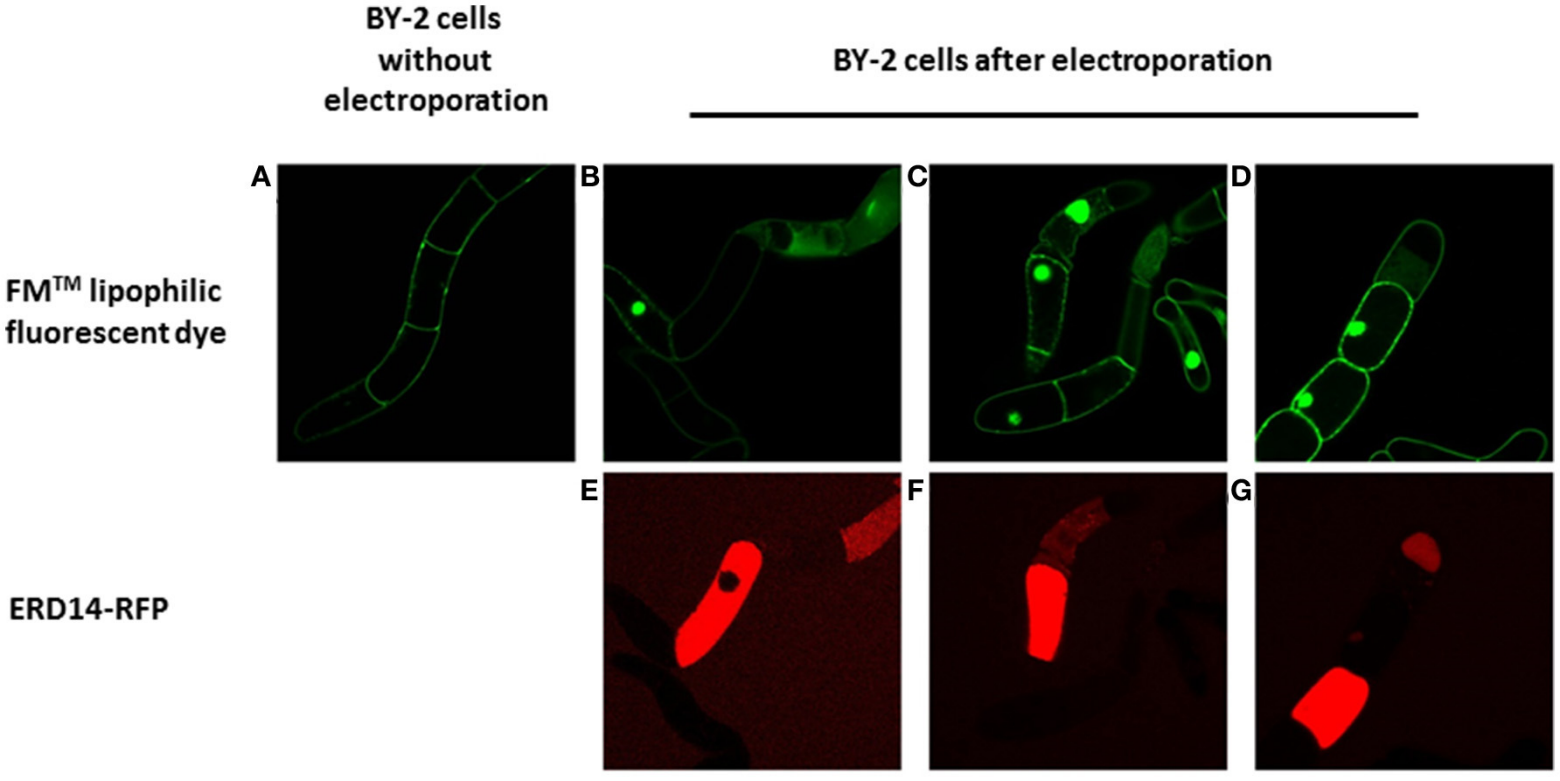
**Fluorescent microscopy analysis of the transient permeabilization of BY-2 cells by electroporation with the lipophilic styryl fluorescent dye FM and ERD14-C186-Alexa Fluor 647**. Normal BY-2 cells before being electroporated **(A)** are not permeable to FM (green). BY-2 cells in which the plasma membrane is disrupted by electroporation **(B–D)** display incorporation of FM that is mainly located at the nucleus. ERD14-C186-Alexa Fluor 647 penetrates the inner space of BY-2 cells after electroporation **(E–G)**.

For this purpose, a great variety of electroporation conditions were tested to identify the mildest one with as small as possible effect on cell viability. In the first instance, we used the fluorescent derivative ERD14- C186-Alexa Fluor 647 to use microscopic visualization to verify if the protein was successfully delivered into the cells. The variables that we scouted in order to explore the delivery/survivability space, are: voltage, capacitance and pulse duration (Table 1). According to the technical notes of the electroporation device, exponential decay represents the best choice in terms of maximizing cell viability during and after the experiment. Only in a few cases we could detect successful delivery of fluorescently labeled ERD14 inside BY-2 cells (Figure 6). However, most electroporation conditions compromised cell viability in such a way that it is not compatible with any downstream *in vivo* experiments. As can be seen in Figure 6, both protein localization and cell morphology strongly indicate non-physiological phenomena. In general, the osmotic balance of cells appears to be heavily disrupted during electroporation (Figure 6). ERD14-C186-Alexa Fluor 647 is distributed homogeneously in the cytoplasm of some cells (Figures 6E–G, red color) showing that most of the inner architecture of the cells is disrupted. Strikingly, as also seen in Figures 6F,G, ERD14-C186-Alexa Fluor 647 is not only distributed across the cytoplasm but it can also be observed inside nuclei. There is no evidence of the association of ERD14-C186-Alexa Fluor 647 with membranes.

These adverse effects do not improve during the recovery period when membrane pores opened by the electric field are supposed to close; it seems that ERD14 can diffuse freely inside cells once pores are opened. Apparently, these set of conditions can break the tonoplast (or vacuolar membrane) whereby the cellular osmotic balance is altered in such a way that is not possible to satisfy both efficient protein delivery and cell viability. Indeed, vacuoles occupy 90–95% of the total internal volume (Marty, 1999) and the pH in the lumen reaches acidic values as low as 5.5 (Barbier-Brygoo et al., 1986). Disruption of the tonoplast is very likely the reason of the uniform distribution of ERD14-C186-Alexa Fluor 647 inside the cells without the preservation of cytosolic strands (Figures 5E–G); this is in striking contrast with the images obtained for healthy cells (Figure 4D). It must be highlighted that the internal morphology shown in this figure is not physiologically relevant as this is accompanied with FM penetration and nuclear staining (Figures 6B–D). FM does not penetrate healthy cells before electroporation (Figure 6A), therefore the conclusion is that most of the cells shown in Figures 6B–D are highly compromised, either because of disruption of vacuoles, or due to the lack of fast and efficient mechanisms repairing plasma membrane pores through cytoskeleton remodeling (Zhang et al., 2015). The observed state as shown in Figure 6 is representative of all the conditions of electroporation tested (Table 1) as there are no differences or trends observed that can be attributed to changes in voltage or intensity.

This observed vacuole disruption by electroporation also offers another interpretation concerning our *in vitro* crowding experiments (Figures 2, 3). These experiments did not show substantial structural changes in cell lysates as compared to a simple *in vitro* buffer system. Cytosolic strands are the regions (Figure 4) where both ERD14 and ERD10 are localized and where total, non-homogeneous (Luby-Phelps, 2013) protein concentration is expected to be at least 200 mg/mL (Theillet et al., 2014). The large volume of water contained in vacuoles (Marty, 1999) dilutes the cytosolic strands up to 20 times, since cell lysis disrupts the plasma membrane as well as tonoplasts. In Figures 2, 3, the crude cell lysate contains 5–7 mg/mL of protein, this low total concentration of proteins is a clear evidence of the dilution effect introduced by of vacuoles. Although the application of cell lysates/extracts is a frequently used approach to approximate or mimic intracellular effects, this might not apply for physical/structural studies where an ideal crowding experiment (such as for plant proteins) needs to be performed at high total protein concentrations like those exhibited inside cells. This offers a pivotal argument to develop a true *in-cell* NMR strategy.

### Induced overexpression of ERD14 in plant cells

**Figure 7:**
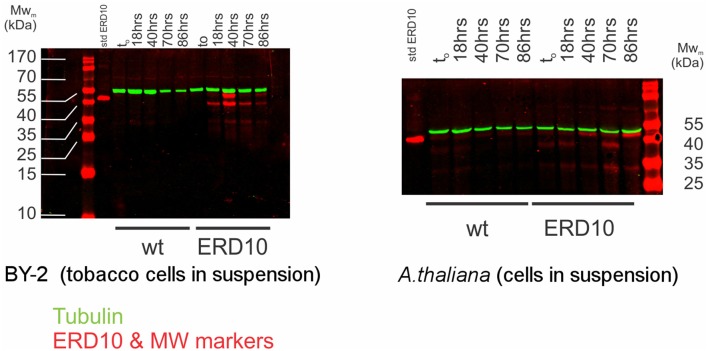
**Estradiol-induced overexpression of ERD10 in BY-2 cells and *Arabidopsis thaliana* cell suspension**. ERD10 gets overexpressed in transformed BY-2 cells upon induction with estradiol as shown by a dual color simultaneous western blotting technique. Recombinantly produced and purified ERD10 was included as a reference for the molecular weight of the native protein without any kind of post-translational modification (std ERD10). Expression of ERD10 was monitored from t_o_ (the point in which estradiol was added) until 86 h. The endogenous tubulin was monitored as a reference to the cell WT refers to samples from non-modified plant cells treated identically as genetically modified plant cells that contain *erd10* under control of the inducible promoter.

While electroporation-mediated introduction of ERD14 did not yield the desired outcome for further *in-cell* NMR studies, we resorted to controlled overexpression of proteins inside plant cells as reported in the past (Hellwig et al., 2004; Ohki et al., 2008). In order to enable the production of 15N-labeled ERD proteins inside either BY-2 or *A. thaliana* cells in suspension, we generated transgenic lines of A. thaliana and BY-2 in which *erd14* and *erd10* genes were controlled by an inducible estrogen promoter. Unfortunately, gene transfection of *erd14* did not yield any viable culture and therefore positive *erd14* cell line could not be obtained in A. thaliana. In the case of BY-2, calli and cell suspension culture were obtained for transformants of both genes (ERD14 and 10), however, only ERD10 gets overexpressed upon the addition of estradiol, as detected by immunoblotting after 40 h of induction (Figure 7).

**Figure 8:**
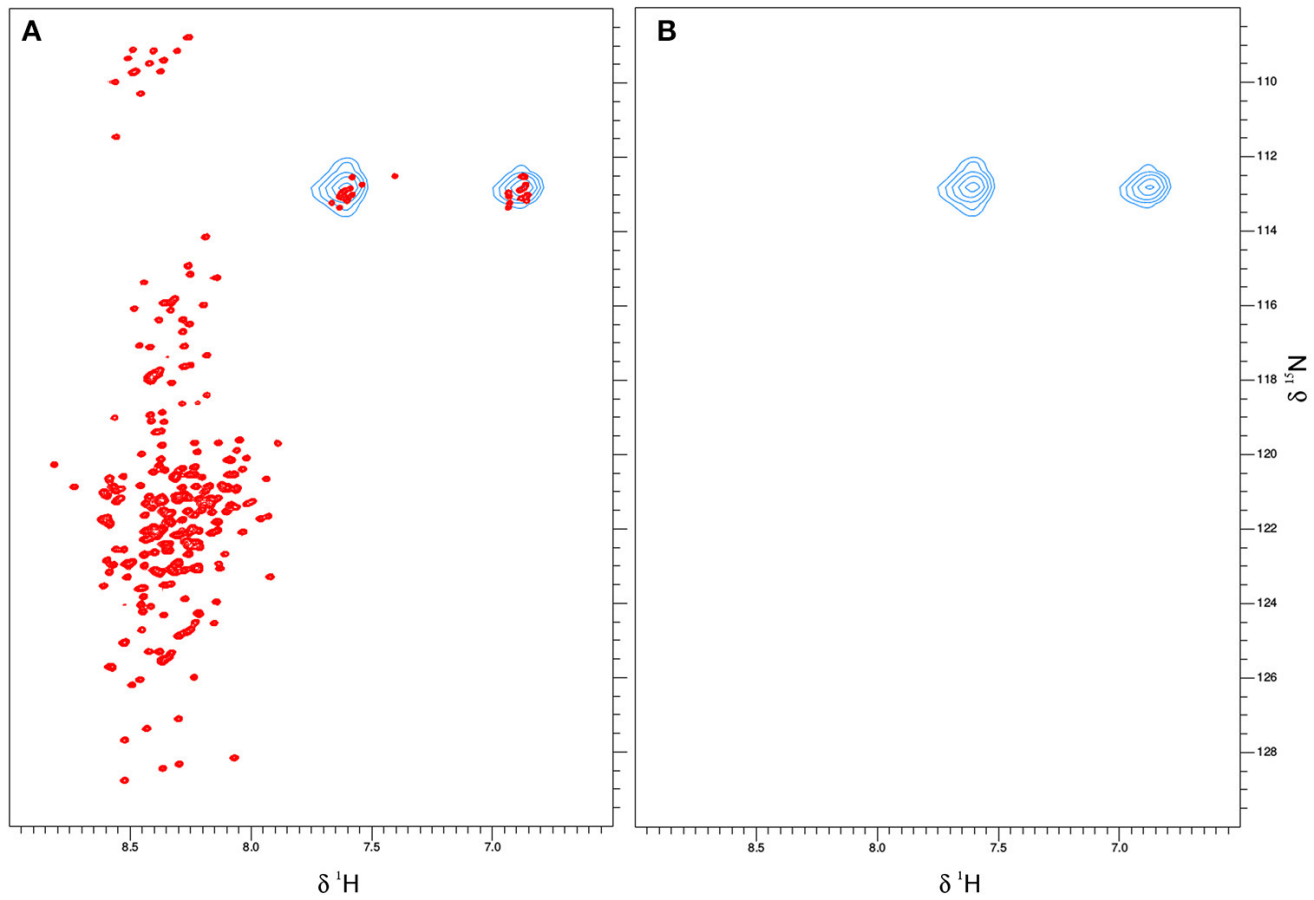
**ERD10 is not detectable by NMR when overexpressed inside plant BY-2 cells**. The ^1^H-^15^N-HSQC spectrum of 100 μM ERD10measured in 50 mM MES buffer pH = 6.5 that is represented by the red cross-peaks is overlayed with ^15^N-labeled glutamine and asparagine side chain signals that are represented as blue contours **(A)**. The ^1^H-^15^N-HSQC spectrum of BY-2 cells transformed with *erd10* under the control of an inducible promoter does not display visible protein cross-peaks after estradiol-induced protein expression and continued growth for 48 h **(B)**. Only the ^15^N-labeled glutamine NH2 side chain signals are detected, which indicate that the ^15^N-source is successfully metabolized into amino acids.

An inducible protein expression system can offer a major advantage over protein delivery via electroporation, because cells are constantly growing under normal culture conditions. Therefore, cell viability is much less affected during expression of ERD10 and thus cultures can grow normally during the progression of the experiment (Figure 7). However, overexpression of ERD10 in BY-2 cells imposes a set of restrictions, primarily the inherent background generated by other endogenous proteins under isotope-labeling conditions. In this scenario, ERD10 needs to be 15N labeled (at least) in order to generate a useful sample for *in-cell* NMR. As exemplified by the *E. coli* overexpression systems, translation needs to be fast and strong to obtain a good signal-to-noise ratio in an *in-cell* ^1^H-^15^N-HSQC experiment (Serber et al., 2001). Liquid media for plant cultures normally contain a set of inorganic salts (micronutrients) in low concentrations and a large supply of carbon in the form of sucrose (besides vitamins and the buffer, known as Murashige-Skoog media). Vitamins like myo-inositol, thiamine and the hormone auxin determines the healthy growth of BY-2 cells, however, the mixture of vitamins and hormones can differ from one species to the other. The source of nitrogen contained in the original Murashige-Skoog medium is changed for *in-cell* NMR samples. In this case they were replaced by 15N-containing salts to make ERD10 visible for NMR upon estradiol-induced protein expression. The first metabolic step once ammonium and nitrate reaches the cytosol is its absorption in the form of glutamine, hence this amino acid is highly abundant in a nitrogen-rich environment (e.g., *in vitro* cultures) (Stitt et al., 2002; Masclaux-Daubresse et al., 2006). As shown in Figure 3 the *in vitro* NMR spectra of ERD10 in buffer and in a cellular extract depict the lower limit of detection of a ^1^H-^15^N-HSQC with an acceptable signal-to-noise ratio given the low concentration and pH differences. Only qualitative information about ERD10 (fingerprint of disorder, post-translational modifications, degradation, etc.) can be extracted from these spectra (Figure 3) although lower amounts of protein would not yield easily interpretable data. These experiments show that about 12.5 μM is the lower limit of ERD10 at which NMR signals can be generated and collected with acceptable good signal-to-noise ratio, which sets our practical limit for the feasibility of the *in-cell* NMR experiments This was not the case in our overexpression experiment: the levels of ERD10 in BY-2 cell cultures after 48 h of induction can rise up to 2.5 μM as semi-quantitatively measured by Western Blot (Figure 7). When we compare the spectrum of a highly concentrated sample of ERD10 overlapped on the spectrum of BY-2 cells containing 2.5 μM ERD10 (Figure 8A), it is clear that sharp and intense signals can be collected from a highly concentrated sample, however, the signals coming from labeled proteins inside BY-2 are not detectable. Instead, two very intense and broad lines are detected in the region of glutamine side chains (Figure 8B), which suggests that BY-2 cells are actively metabolizing nitrate and incorporating ^15^N, although the translation products of other proteinaceous species including ERD10 are not detectable. This observation is in accordance with quantification of protein levels by Western blot.

## Discussion

As the *modus operandi* and functional behavior of intrinsically disordered ERD14 and ERD10 in protection against abiotic stress remain elusive, we set out to develop an *in-cell* NMR strategy with plant cells. First, we established by fluorescent fusion proteins that under our experimental conditions ERD14 and ERD10 are cytosolic proteins that do not change their intracellular localization under abiotic stress conditions (cold and osmotic stress). Next, by simulating the cellular crowding using cell extracts we evaluated that ERD14 and ERD10 are compatible with an NMR read-out to study molecular events inside cells (Smith et al., 2016). Whereas the use of cellular extracts has been considered as a reasonable mimic for intracellular conditions and protein behavior, we observed that in the case of plant cell extracts the molecular crowding and excluded volume effect are limited and inappropriate, due to a significant dilution of the cytosol by the vacuolar content. Thus, *in-cell* NMR actually creates a particular opportunity to understand protein structure and activity from a unique perspective.

There are several considerations and strategies for protein delivery for the purpose of *in-cell* NMR (see Figure 1) and the motivations and methodological approaches can be manifold. Further, there are diverse controls and additional experiments to carry out for exploiting the full potential of this technology. However, major barriers imposed by physiology of the plant cell can restrict its applicability in many ways. There are limitations to the scenario in which *in-cell* NMR is feasible: cells must survive the treatment prior to the insertion in an NMR tube, the physiology should not be altered in such a way that cellular morphology is dramatically affected and the NMR spectra of proteins (isotopically labeled) should be collected at intracellular concentrations close to the physiological ones (in un-treated cells). Electroporation was selected as our preferred delivery method during the initial stages of this study, because pore formation evoked by pulsating electric fields yielded good results for the transformation of both prokaryotic and eukaryotic cells (Kato et al., 2010; Boukany et al., 2011; Wang et al., 2014; Theillet et al., 2016). Microinjection cannot be applied because the size and morphology of tobacco cells (BY-2) represented a major challenge (due to the size of its vacuoles and also because microinjection is mostly only useful with larger cells (e.g., oocytes).

Vacuoles are dynamic and mechanically resistant in order to perform their function without a loss of integrity, as they are responsible for maintaining the osmotic pressure, pH and membrane potential of the cell. Strong depolarizations and dramatic changes in pH or osmolarity beyond the vacuolar capability are life-threatening for the cell. Basically, electroporation is a strong depolarization able to generate a transient permeabilization of the plasma membrane as has been evidenced in gene transfection (Weaver, 1995; Ho and Mittal, 1996). However, in light of the current evidence, electroporation also generates a transient disruption of tonoplasts (that are enriched in acidic phospholipids and in sterols (Zhang et al., 2015), which likely explains why cells become compromised with their internal cell morphology heavily disrupted. Despite varying electroporation conditions (Table 1), ERD14 could only enter cells in which survivability was compromised, with large amounts of cells not showing signs of either damage or protein delivery. This is likely the consequence of a strong protective effect of the plant cell wall, suggesting that internal membranes can no longer survive the electric shock once the protective cell wall is brought down in this time scale (micro-to-millisecond of electric pulse) (Batista Napotnik et al., 2016). Altogether, these results show limitations of electroporating plant cells with the particular aim of performing *in-cell* NMR.

To overcome these obstacles, we also approached the problem by induced overexpression under isotope- labeling conditions. Unfortunately, the levels of protein expression are not compatible with the low sensitivity of the state-of-the-art technique. For each protein the optimal expression conditions (i.e., NMR signal intensity as compared to the background signals that arise from non-specific isotopic enrichment during the expression of endogenous proteins) should be screened and quantitative Western Blotting of cell lysates at different expression times or with different promoters is a reliable technique to accomplish this (Figure 1). This strategy also opens the possibility to co-express partner proteins. The choice of cell line should also be considered carefully: in this work we used BY-2 cells and *Arabidopsis* cells in suspension. An interesting alternative that would be compatible with *in-cell* NMR would be the unicellular green algae *Chlamydomonas*, but this would not be the primary choice to study phenomena related to dehydration stress (Yamano et al., 2013).

For the future, other methodologies should be considered for the gentle manipulation of cells in order to introduce isotope-labeled reporter proteins. These methodologies can exploit the machinery of exogenous agents (bacteria and viruses) in order to penetrate and deliver products selectively inside the cytoplasm. For example, bacterial toxins like streptolysin can be used for the purpose of *in-cell* NMR (Ogino et al., 2009), however, to the best of our knowledge, there are no reports of its use on plants. Some bacterial pathogens actively translocate their virulence factors using a molecular needle that is able to penetrate membranes, e.g., the type III and type IV secretion system (t3ss, Galan et al., 2014; and t4ss, Fronzes et al., 2009). Even though this is a widespread mechanism for bacterial protein delivery in nature, there are limitations for its use toward *in-cell* NMR. Basically, proteins delivered using t3ss are typically of low abundance and the pathogens exploiting this invasion mechanism have not evolved for larger loads in terms of concentration. *A. tumefaciens* infects plants using t4ss and this is the key mechanism for gene introduction. In terms of synthetic biology and engineering, t3ss and t4ss are interesting tools but for *in-cell* NMR, it needs to be coupled with an efficient inducible overexpression system, besides the proper translocation signals on bacteria. The whole approach represents a largely unexplored field of research that is expected to bring exciting results in the future.

An interesting alternative for translocation and delivery of cargo proteins of even larger size into cells are cell-penetrating peptides (CPPs). These small peptides were already successfully exploited by NMR spectroscopists to explore the interior of mammalian cells (Inomata et al., 2009). CPPs, however, are not yet used at large scale for in-cell NMR, most likely due to their tendency to stay trapped in endosomes dragging the cargo protein with them (Nischan et al., 2015). Nonetheless, CPPs constitute an active field of research that is of particular interest for the pharmaceutical industry and they are intensely studied from a technological and methodological point-of-view. In plants, they proved to be useful for delivering proteins into the cytosol as shown by Herce et al. (2014) and more recently by Ng et al. (2016). Therefore, these CPPs are a very promising research avenue that hold promise to be exploited for studying ERD14 or ERD10 structure and function inside living plant cells through in-cell NMR.

## Author contributions

CC and PT contributed to the conception and design of the work. CC was responsible for the acquisition of the data presented in this work. CC and KP performed the analysis and interpretation of the data. All authors contributed to the writing of the paper.

## Funding

CC was supported by the Marie Curie Initial Training Network project 264257 (IDPbyNMR). PT was supported by the Odysseus grant G.0029.12 from Research Foundation Flanders (FWO). KP is the recipient of a FWO long-term postdoctoral fellowship (1218713). The funders had no role in study design, data collection and analysis, decision to publish, or preparation of the manuscript.

### Conflict of interest statement

The authors declare that the research was conducted in the absence of any commercial or financial relationships that could be construed as a potential conflict of interest.
